# No evidence for altered plasma NGF and BDNF levels in fibromyalgia patients

**DOI:** 10.1038/s41598-019-49403-7

**Published:** 2019-09-20

**Authors:** David Baumeister, Wolfgang Eich, Silvia Saft, Olga Geisel, Rainer Hellweg, Anja Finn, Camilla I. Svensson, Jonas Tesarz

**Affiliations:** 1 Department of General Internal Medicine and Psychosomatics, University Hospital, Heidelberg, Germany; 2 Department of Psychiatry and Psychotherapy, Charité, Campus Mitte, Berlin, Germany; 3 Department of Physiology and Pharmacology, Center for Molecular Medicine, Karolinska Institute, Stockholm, Sweden

**Keywords:** Fibromyalgia, Chronic pain

## Abstract

There has been a surging interest in the putative role of peripheral growth factors in the pathophysiology of fibromyalgia, specifically in the peripheral sensitization that occurs in chronic pain disorders. This cross-sectional study set out to assess and compare brain-derived neurotrophic factor (BDNF) and nerve growth factor (NGF) in plasma samples from fibromyalgia patients and healthy controls. Plasma BDNF and NGF were measured in 89 fibromyalgia patients and 36 pain-free controls, and compared using ANCOVA controlling for potential confounders, as well as Bayesian methods for parameter estimation and model evaluation. BDNF and NGF levels in fibromyalgia patients did not differ from those in pain-free controls. Statistical methods were consistent, with both frequentist and Bayesian approaches leading to the same conclusions. Our study fails to replicate the finding that peripheral BDNF is altered in fibromyalgia, and instead our findings suggest that plasma levels of growth factor appear normative in fibromyalgia.

## Introduction

Fibromyalgia is a chronic musculoskeletal pain condition marked by widespread pain^1,2^. Fibromyalgia is one of the most pervasive primary pain syndromes, affecting 2–5% of the general population^3^. It has been proposed that both alterations in central and peripheral sensitization partially underlie the development of the disorder^4^. In order to identify biomarkers that may explain central and peripheral sensitization at the molecular level, research has turned to growth factors, including brain-derived neurotrophic factor (BDNF) and nerve growth factor (NGF). Excessive activity of these markers has been posited to drive sensitization, and a maladaptive role for BDNF and NGF has been highlighted in preclinical evidence^4,5^. Indeed, there are reports that BDNF^6^ and NGF^6,7^ are increased in the cerebrospinal fluid of fibromyalgia patients. Whilst growth factors have primarily been hypothesized to exert sensitizing effects at central and spinal levels, several studies have reported that that peripheral BDNF is increased in fibromyalgia^8–11^, and that it correlates with altered pain pressure threshold^12^. However, there are also reports of normative BDNF serum levels^13^, and to the best of our knowledge there are no published data on peripheral NGF levels in fibromyalgia patients. Thus, the utility of neurotrophic biomarkers in fibromyalgia remains unclear.

For the present study we investigated plasma levels of NGF, as well as BDNF as a replication attempt in a large sample of fibromyalgia patients. We hypothesized increased levels of BDNF and NGF in fibromyalgia patients as compared to pain-free controls, indicative of peripheral sensitization in the chronic pain population.

## Methods

This study was part of the research consortium “Localized and Generalized Musculoskeletal Pain: Psychobiological Mechanisms and Implications for Treatment” (LOGIN), funded by the German Federal Ministry of Education and Research (01EC1010A-F) and reported on recently^14–16^. All participants provided written informed consent before their inclusion in the study. The Ethics Research Committee of the Medical Faculty, University of Heidelberg approved the study (S-261/2010), which was performed in accordance with the Helsinki Declaration.

### Sample

For the present study, 97 fibromyalgia patients were recruited from a tertiary care pain center at the University Hospital Heidelberg, as well 35 pain-free controls. To be included, fibromyalgia patients had to be at least 18 years of age, not suffer from specific pathologies, and not take antipsychotics, benzodiazepines or prescription opiates. In addition, participants were advised not to take any pain medication 24 hours prior to the investigation. Fibromyalgia status was confirmed using the 1990 American College of Rheumatology criteria, according to which participants had to report chronic widespread pain (i.e., chronic contralateral limb pain) with at least 11 of 18 positive tender points. Symptoms had to be present for ≥45 days of the past 3 months to ensure clinically relevant chronicity^15^. See our previous studies on this sample^14,15,17^ for a more comprehensive description of inclusion and exclusion criteria. Pain-free controls were recruited through opportunity sampling and subjected to the same inclusion criteria, plus the absence of any chronic pain condition.

### Measures

All participants completed a sociodemographic assessment capturing age, gender, marital status, level of education and employment. Additionally, average pain intensity and pain days were reported for the previous 4 weeks. Pain intensity was rated using a numerical rating scale ranging from 0 (“no pain”) to 10 (“worst pain imaginable”). All participants completed the Hospital Anxiety and Depression Scale (HADS) to determine the severity of anxiety and depressive symptoms^18^. In the present study, the HADS showed reliability of the 7-item depression (range 0–21; Cronbach’s α = 0.85) and the 7-item anxiety (range 0–21; Cronbach’s α = 0.82) subscales. All participants underwent venepuncture to assess peripheral growth factors. We used 2.7 ml EDTA vacutainers. Bloods were immediately (<1 min) centrifuged at 4 °C at 2000 g for 10 min, plasma was aliquoted and frozen in liquid nitrogen. An immunoassay for analysis of NGF was developed using a prototype based on electrochemiluminiscense (ECL) technology (Mesoscale Discovery, Rockville, MD, USA). All samples were measured in duplicate. The plates (96- well format) were preprocessed by the manufacturer with a specific antibody directed towards human NGF. Recombinant human beta NGF was used as a calibrator (#256-GF, R&D systems, Minneapolis, MN, USA). Equal volumes of diluent and sample were added to the wells and incubated for 2 hrs. After a washing step a biotinylated detection antibody provided with MSD Sulfo TAG was dispensed to all wells and incubated for another 1.5 hrs. Plates were subsequently washed and read buffer was added prior to analysis on the SECTOR Imager. Lower limit of quantification was 0, 61 pg/mL. Intra- and interassay coefficient of variation were 10% and 19% respectively. BDNF was measured in assay buffer diluted serum using commercial fluorometric enzyme-linked immunosorbent assay kits (Promega Inc., Mannheim, Germany) as described previously^19,20^.

To control for variations in dietary behaviours and health, body-mass index (BMI), time since last meal, time of day at venepuncture, diabetes mellitus, hours of sleep the previous night, as well as recent consumption of coffee, chocolate and spicy foods were recorded. We further recorded current medications, including antidepressants, ion channel blockers (Na, Ca, Ka), opiates and NSAIDs.

### Statistical analysis

All statistical analyses were carried out in R (3.4.1). To maximise confidence in statistical findings, both frequentist as well as Bayesian statistical models were calculated. For descriptive statistics, t-tests and chi-square statistics were used. Power analysis using G*Power^21^ indicated that the present sample was sufficiently powered to find effect sizes above Cohen’s d = 0.56. All biomarkers were log-transformed to ensure normal distribution. For frequentist methods, ANCOVA were carried out. Associations with potential covariates were carried out using simple regressions for continuous variables and logistic regression for categorical variables. To allow for conservative model estimations, all ANCOVAs were run twice, once excluding variables significantly different between groups, and once including those variables. If no statistically significant differences between the resulting models were observed, the “classic” model excluding divergent variables is reported. The potential covariates assessed were age, gender, cigarettes per day, grams of alcohol per day, time since last meal, body-mass index (BMI), diabetes mellitus, hours of sleep the previous night, time of day at sampling, level of anxiety and depression, average pain in the last 4 weeks, medication status and basic demographics.

Using Bayesian methods to express posterior probabilities of the data, Bayes Factors (BF_10_) as well as Highest Density Intervals (HDIs) with default priors using Monte Carlo Markov Chains were calculated using the *BayesFactor*, *JAGS* and *BEST* packages. Bayes factors of 3 and above were interpreted as sufficient evidence for group differences, and Bayes factors of 1/3 and below as sufficient evidence for no group differences^22^. Monte Carlo Markov Chain converge was verified using the Potential Scale Reduction Factor^11^. Effect sizes are expressed in Cohen’s d.

## Results

### Sample characteristics

Descriptive statistics are presented in Table 1. Fibromyalgia patients were significantly more likely to be younger, female, have higher BMI, consume less alcohol, and have slept fewer hours the previous night. They had lower educational attainments but similar levels of employment. As would be expected, they also had more pain days in the previous four weeks, with greater average pain, and they were more likely to take antidepressants, channel blockers and NSAIDs.

**Table 1:** Descriptives of the sample populations.

Variable	Fibromyalgia (n = 97)	Healthy Controls (n = 35)	Statistics
Age in years (M (SD))	56.5 (10.0)	61.9 (12.5)	t = 2.6, p = 0.01
Female sex, % (N)	90.7% (88)	42.9% (15)	Χ^2^ = 34.4, p < 0.001
Body mass index in kg/m^2^	29.1 (6.4)	26.8 (4.0)	t = 2.4, p = 0.02
Diabetes mellitus	11.2% (10)	2.8% (1)	Χ^2^ = 2.3, p = 0.1
Cigarettes/day	1.9 (5.6)	1.9 (6.2)	t = 0.03, p = 0.097
Alcohol g/day	1.8 (4.5)	11.6 (15.7)	t = 3.2, p = 0.003
Anxiety (HADS)	9.0 (4.2)	3.6 (2.6)	t = 8.8, p < 0.001
Depression (HADS)	8.0 (4.2)	3.0 (3.3)	t = 7.2, p < 0.001
Average pain/4 weeks (NRS)	6.2 (1.7)	0.0 (0.0)	t = 38.2, p < 0.001
Number of painful areas	8.2 (1.4)	0.1 (0.7)	t = 44.4, p < 0.001
ADs	39.2% (38)	0.0% (0)	Χ^2^ = 19.3, p < 0.001
Opiates	6.2% (6)	0.0% (0)	Χ^2^ = 2.3, p = 0.1
NSAIDs	57.7% (56)	14.3% (5)	Χ^2^ = 19.5, p < 0.001
Channel Blockers	14.4% (14)	0.0% (0)	Χ^2^ = 5.7, p = 0.01
Relationship status (% single)	24.7% (24)	28.6% (10)	Χ^2^ = 0.2, p = 0.7
Education (% > 10 y in school)	17.5% (17)	48.6% (17)	Χ^2^ = 13.0, p < 0.001
Employment status			Χ^2^ = 1.7, p = 0.6
*In employment*	28.9% (28)	40.0% (14)	
*Unemployed*	7.2% (7)	5.7% (2)	
*Retired*	50.5% (49)	45.7% (16)	
*Other*	12.1% (16)	8.6% (3)	

AD: antidepressants, HADS: Hospital Anxiety and Depression Scale; M: mean; NRS: Numerical Rating Scale; NSAID: non-steroidal anti-inflammatory drugs; SD: standard deviation; %: percentage; g: gram; y: years.

### Growth factors

In the case of NGF (reported in pg/mL plasma), ANCOVA controlling for cigarette smoking identified no significant group differences (F(1, 131) = 0.14, p = 0.71). Bayesian analyses suggested no significant differences, with a BF10 of 0.22 and similar mean and HDI estimates for the probable parameter values in fibromyalgia patients (m = 0.66, 95%-HDI = 0.61–0.71) and healthy controls (m = 0.71, 95%-HDI = 0.64–0.78). The mean difference was estimated at −0.06 (95%-HDI = −0.03–0.14; Cohen’s d = 0.08).

**Figure 1:**
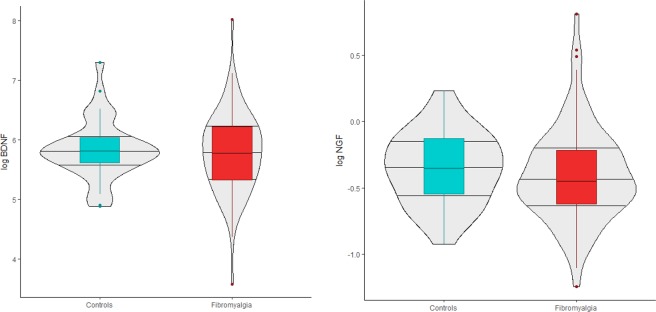
Violin-box plots for growth factors by group.

For BDNF (reported in pg/mL plasma), ANCOVA controlling for channel blockers no significant group differences (F(1, 131) = 0.0, p = 0.995). Bayesian analyses suggested no significant differences, with a BF10 of 0.22 and similar mean and HDI estimates for the probable parameter values in fibromyalgia patients (m = 331.90, 95%-HDI = 287.30–375.84) and controls (m = 326.95, 95%-HDI = 282.50–371.96). The mean difference was estimated at −4.93 (95%-HDI = −68.28–55.62; Cohen’s d = 0.09). Results for BDNF and NGF are presented in Fig. 1.

## Discussion

### Findings

Contrary to the study hypotheses, fibromyalgia patients were indistinguishable from pain-free controls on both plasma NGF and BDNF levels. Our findings therefore do not suggest that peripheral growth factors levels are not a pathophysiological feature of fibromyalgia, or that they contribute to the development of peripheral pain sensitisation.

The likely explanations of these findings are two-fold. First, heterogenous nature of fibromyalgia makes generalisation difficult. Phenotypical phenomena such as central sensitisation may be more easily established than underlying endophenotypical mechanisms of such phenomena. Second, previous studies measuring BDNF had typically less than half the sample size of this study and typically assessed a range of biomarkers, and thus suffered from greater likelihood of chance findings. By concentrating on only growth factors in a large sample of well characterised patients, our study was able to show that they are unlikely to be systematically increased or altered. Given that the extant literature broadly implicates BDNF as a biomarker in fibromyalgia, and further hypothesises NGF to be implicated in the peripheral pathophysiology of fibromyalgia, our null findings will adjust how clear the current study consensus appears.

### Strengths and limitations

Several strengths of the present study can be noted. To our knowledge, this is the first study to assess peripheral NGF in a large sample of fibromyalgia patients and pain-free controls. We further employed rigorous methods to ensure the sample is well defined and characterized and blood samples deliver accurate measurements.

The most marked study limitation is that no cerebrospinal fluid measures of BDNF and NGF were taken, and thus no conclusions can be drawn as to whether central growth factor levels are altered in fibromyalgia. However, it should be noted that BDNF can efficiently cross the blood-brain barrier^23^. Second, heterogeneity between the clinical and control groups may have also skewed reliability of our findings, as there were significant group differences in age, gender and important psychosocial variables such as medications or alcohol consumption. However, these were not identified as potential covariates, suggesting they did not influence growth factor levels.

### Implications and future directions

The present study findings contradict those from previous studies measures BDNF in blood of fibromyalgia patients, and this finding should be explored further. Future research should further utilise cerebrospinal fluid to seek whether central growth factors levels are similarly unaltered, and how these levels correspond to those in the periphery. Further, the association to peripheral and central sensitisation and growth factors should be investigated.

## Conclusions

This is the first study to show that peripheral NGF is unperturbed in fibromyalgia patients, and further sheds doubt upon previous findings that plasma BDNF is increased in fibromyalgia.

## Data Availability

Anonymised parts of the data can be made available upon request.
